# Adverse neurodevelopment after multiple sepsis and/or necrotizing enterocolitis in preterm infants: revisiting single-episode paradigm

**DOI:** 10.1038/s41390-025-04102-0

**Published:** 2025-06-07

**Authors:** Jae Hui Ryu, Seung Han Shin, Baek Sup Shin, Ee-Kyung Kim, Han-Suk Kim

**Affiliations:** 1 Department of Pediatrics, Ewha Womans University College of Medicine, Ewha Womans University Mokdong Hospital, Seoul, South Korea; 2 Department of Pediatrics, Seoul National University College of Medicine, Seoul National University Children’s Hospital, Seoul, South Korea

## Abstract

**Background:**

Systemic inflammation in preterm infants is associated with an increased risk of adverse neurodevelopmental outcomes. This study aimed to investigate the impact of single versus multiple episodes of sepsis and/or necrotizing enterocolitis (NEC) on neurodevelopmental impairment (NDI) in this population.

**Methods:**

This cohort study used data from a nationwide registry, including very low-birth-weight infants born before 32 weeks of gestation from 2013 to 2020. The study population was categorized according to the occurrence of sepsis and/or NEC. Neurodevelopmental assessments at 18–24 months of corrected age were performed using various tools. Any NDI or death was used as the primary outcome.

**Results:**

In the multivariate logistic regression analysis, infants with multiple episodes of sepsis (aOR = 1.43; 95% CI [1.02–2.01]) or both sepsis and NEC (aOR = 1.91; 95% CI [1.26–2.90]) had a significantly higher risk of NDI compared to those without sepsis and NEC. A single sepsis episode without NEC was not associated with an increased risk of NDI.

**Conclusion:**

Multiple episodes of sepsis and/or NEC significantly increased the risk of NDI in VLBW infants, whereas a single episode of sepsis did not. These findings highlight the need to distinguish between single and multiple episodes of systemic inflammation when assessing neurodevelopmental outcomes.

**Impact:**

- Multiple episodes of sepsis and/or necrotizing enterocolitis (NEC) significantly increase the risk of neurodevelopmental impairment (NDI) and death in preterm infants. However, a single episode of sepsis alone was not associated with the risk of NDI and NDI or death in the study population. When evaluating the neurodevelopmental outcomes of preterm infants, it is crucial to recognize that a single episode of sepsis may have a lesser impact on NDI compared to recurrent systemic inflammation or NEC episodes.

## Introduction

Preterm infants are at an increased risk of impaired neurodevelopment, with adverse outcomes associated not only with evident brain injury but also with other morbidities experienced during the neonatal period.^1–3^ Numerous perinatal and postnatal factors have been implicated in these outcomes, with a notable emphasis on conditions that induce systemic inflammation, such as sepsis and/or necrotizing enterocolitis (NEC).^4–6^ The association of systemic inflammation with adverse neurodevelopmental outcomes is linked because pre-myelinating oligodendrocytes are vulnerable to various insults, including hypoxia, ischemia, and inflammatory cytokines, which subsequently lead to white matter injury in preterm infants.^6,7^

In contrast, several studies have reported no discernible association between sepsis and neurodevelopmental impairment (NDI) in preterm infants.^8–10^ To address this gap, it should be noted that some preterm infants experience systemic inflammation for more than a single episode.^11^ Data from the National Institute of Child Health and Human Development (NICHD) Neonatal Research Network revealed that 12.7% of very low birth weight (VLBW) infants encounter both sepsis and NEC during their hospitalization.^4^ On the other hand, of patients who experience NEC, approximately 30–60% also suffer from sepsis.^12,13^ Therefore, to measure the effect of systemic inflammation episodes on neurodevelopment, it is necessary to distinguish between single and multiple episodes of systemic inflammation.

This study aimed to provide further insights by analyzing the impact of systemic inflammatory events on NDI in VLBW infants and categorizing them into those experiencing a single episode and those experiencing multiple episodes of systemic inflammation. Our objective was to elucidate whether a single episode of sepsis alone is associated with NDI and whether the risk of NDI increases in the presence of multiple episodes.

## Methods

### Study population

The Korean Neonatal Network (KNN) is a nationwide prospective registry of VLBW infants born in the Republic of Korea. The data registered in the database comprise antenatal and perinatal factors and postnatal morbidities evaluated during the hospital stay and developmental outcomes after discharge using a standardized electronic case report form. Among those registered in the KNN database from January 2013 to December 2020, infants born at less than 32 weeks of gestation were enrolled in this study. Cases were excluded from the study population if they were reported in the database as having congenital anomalies, congenital infections, or meningitis. Patients without data on sepsis or NEC were also excluded. The study population was categorized as follows: those without any episode of sepsis or NEC (Sepsis−/NEC−), those with a single episode of sepsis without NEC (Sepsis+/NEC−), those with NEC without sepsis (Sepsis−/NEC+), those with multiple episodes of sepsis without NEC (Sepsis++/NEC−), and those with sepsis and NEC during the neonatal period (Sepsis+/NEC+).

The Institutional Review Board of Seoul National University Hospital exempted this study from review, as the study involved the use of de-identified registered data (IRB No. 2303-012-1409). This study was conducted in accordance with the principles of the Declaration of Helsinki and the STROBE reporting guidelines.

### Definitions

Neurodevelopmental assessments at 18–24 months of corrected age (CA) in this study involved various tools because of its multicenter nature, including the Bayley Scales of Infant Development II and III (BSID II & III), the Korean Ages and Stages Questionnaire (K-ASQ), and the Korean Developmental Screening Test (K-DST).^14,15^ Mental developmental delay was defined as meeting any of the following criteria: a mental developmental index under 70 in the BSID II; cognitive or language scores below 70 in the BSID III; communication or problem-solving scores below the threshold in the K-ASQ; or below-threshold scores in cognition, language, or self-help in the K-DST.

Motor developmental delay was defined as a psychomotor developmental index of <70 in the BSID II, a motor score of <70 in the BSID III, gross or fine motor scores below the cut-off in the K-ASQ or K-DST, or a diagnosis of cerebral palsy with a Gross Motor Functional Classification System score of ≥2.^16^ Blindness was defined as the presence of either unilateral or bilateral visual impairment, whereas hearing loss was defined as the presence of either unilateral or bilateral auditory impairment. Based on the definitions provided, the presence of any condition, including blindness, hearing loss, mental or motor delay, was classified as having NDI. The primary outcome was a composite of any NDI or death at 18–24 months of CA.

Sepsis was defined as the presence of bacteria in the blood culture, and instances with two or more independent events were defined as “multiple episodes of sepsis.” NEC was defined as Bell’s stage II or III and was diagnosed clinically or radiologically.^17^ Severe retinopathy of prematurity (ROP) was defined as ≥stage 3.^18^ Severe intraventricular hemorrhage (IVH) was defined as the presence of grade 3 or 4 IVH, and periventricular leukomalacia (PVL) was defined after being identified on brain imaging.^19^

### Statistical analysis

The chi-squared test was used to compare categorical variables, while analysis of variance was used to analyze continuous variables among various groups. The Bonferroni correction was used to determine the statistically significant group differences (*p* < 0.005). The relationship between the study group and primary outcome, as well as neurodevelopmental outcomes, was examined using multivariate logistic regression analyses, accounting for gestational age, small for gestational age status, sex, histologic chorioamnionitis, and postnatal morbidities, such as moderate-to-severe bronchopulmonary dysplasia (BPD), severe IVH, PVL, and severe ROP, as possible confounders. The listwise deletion method was employed to address the issue of missing data in our analysis. A sub-cohort analysis was performed after excluding infants with sepsis to examine the association of medical and surgical NEC without sepsis with NDI or death. Data are presented as numbers (%), median, or mean ± standard deviation, and all statistical analyses were performed using R (version 4.0.2, R Foundation for Statistical Computing, Vienna, Austria). The level of statistical significance was set at *p* < 0.05.

## Results

**Fig. 1:**
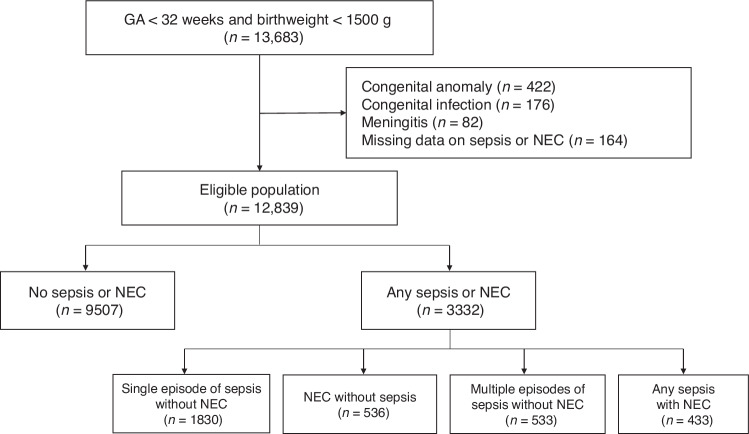
Flow chart of the study population. A total of 12,839 infants with gestational age < 32 weeks and birth weight < 1500 g were included in the study.

Among the infants registered in the KNN database during the study period, 13,683 were born before 32 weeks of gestation. Of these, 422 infants with congenital anomalies, 176 with congenital infections, and 82 with cases reported as meningitis in the KNN database were excluded from the study (Fig. 1). Furthermore, 164 infants with missing sepsis or NEC data were excluded. Of the remaining 12,839 infants, 1830 (14.3%) had a single episode of sepsis without NEC (Sepsis+/NEC−), 536 (4.2%) had NEC without sepsis (Sepsis−/NEC+), 533 (4.2%) had multiple episodes of sepsis without NEC (Sepsis++/NEC−), and 433 (3.4%) had both sepsis and NEC (Sepsis+/NEC+).

Infants with sepsis and/or NEC were born at a lower gestational age than those without sepsis or NEC (Sepsis−/NEC−) (Table 1). The proportion of histologic chorioamnionitis was higher in infants who experienced multiple sepsis events compared to those with no events or a single event of sepsis or NEC. Among all postnatal morbidities—including surfactant use, moderate-to-severe BPD, IVH, PVL, ROP, and death—the Sepsis−/NEC− group consistently had the lowest rates. The rates of moderate-to-severe BPD and ROP were significantly higher in the Sepsis++/NEC− and Sepsis+/NEC+ groups compared to the Sepsis+/NEC− and Sepsis−/NEC+ groups. The incidence of IVH and PVL was lower in the Sepsis+/NEC− group than in the other three groups. The proportion of deaths was significantly higher in infants who experienced NEC (Sepsis−/NEC+, Sepsis+/NEC+) than in those who did not (Sepsis+/NEC−, Sepsis++/NEC−).

**Table 1:** Demographics of the study population according to the study groups

	Sepsis−/NEC− (*n* = 9507)	Sepsis+/NEC− (*n* = 1830)	Sepsis−/NEC+ (*n* = 536)	Sepsis++/NEC− (*n* = 533)	Sepsis+/NEC+ (*n* = 433)	*p*-value
GA (week)	28.4 ± 2.2^§¶†‡^	27.3 ± 2.2^*¶†‡^	26.9 ± 2.2^*§†‡^	26.4 ± 2.0^*§¶^	26.1 ± 2.1^*§¶^	<0.001
Birth weight (g)	1084.5 ± 274.2^§†‡^	954.1 ± 266.3^*¶‡^	893.8 ± 272.9^‡^	849.2 ± 246.1^*§^	823.2 ± 237.8^*§¶^	<0.001
SGA	936 (9.8%)^¶‡^	211 (11.5%)	84 (15.7%)^*^	73 (13.7%)	62 (14.3%)^*^	<0.001
Male	4783 (50.3%)	925 (50.5%)	279 (52.1%)	285 (53.5%)	256 (59.3%)	0.004
Cesarean section	7418 (78.0%)	1424 (77.8%)	420 (78.4%)	394 (73.9%)	312 (72.1%)	0.011
Multiple birth	3508 (36.9%)	652 (35.6%)	179 (33.4%)	170 (31.9%)	146 (33.7%)	0.050
hCAM	2958 (36.5%)^†^	623 (40.0%)^†^	172 (38.1%)^†^	210 (48.2%)^*§¶^	147 (40.7%)	<0.001
Antenatal steroid	8001 (85.1%)	1553 (85.6%)	444 (85.4%)	445 (85.6%)	361 (86.0%)	0.966
Maternal education (college or higher)	5462 (75.9%)	1031 (74.5%)	302 (75.9%)	319 (76.0%)	243 (70.8%)	0.244
Apgar score 1 min	5 (3)	4 (3)	4 (2)	4 (3)	4 (3)	
Surfactant use	7991 (84.1%)^§¶†‡^	1713 (93.6%)^*^	492 (91.8%)^*†‡^	515 (96.6%)^*¶^	419 (96.8%)^*¶^	<0.001
Moderate-to-severe BPD	2410 (28.5%)^§¶†‡^	794 (52.0%)^*†‡^	198 (53.8%)^*†‡^	348 (75.0%)^*§¶^	234 (76.0%)^*§¶^	<0.001
IVH (grade ≥ 3)	689 (7.5%)^§¶†‡^	247 (13.8%)^*¶†‡^	111 (21.1%)^*§†‡^	108 (20.3%)^*§¶^	100 (23.3%)^*§¶^	<0.001
PVL	643 (7.0%)^§¶†‡^	197 (11.0%)^*¶†‡^	72 (13.8%)^*§^	78 (14.7%)^*§‡^	74 (17.4%)^*§†^	<0.001
ROP (stage ≥ 3)	851 (10.0%)^§¶†‡^	306 (19.7%)^*†‡^	86 (22.2%)^*†‡^	163 (34.0%)^*§¶^	116 (35.2%)^*§¶^	<0.001
Death	1017 (10.7%)^§¶†‡^	311 (17.0%)^*¶‡^	175 (32.6%)^*§†^	99 (18.6%)^*¶‡^	159 (36.7%)^*§†^	<0.001

Values are expressed as *N* (%), means ± standard deviations or median (interquartile range).

*Sepsis−/NEC−* No episode of sepsis or necrotizing enterocolitis (NEC), *Sepsis+/NEC−* single episode of sepsis without NEC, *Sepsis−/NEC+* NEC without sepsis, *Sepsis++/NEC−* multiple episodes of sepsis without NEC, *Sepsis+/NEC+* both sepsis and NEC, *GA* gestational age, *SGA* small for gestational age, *hCAM* histologic chorioamnionitis, *BPD* bronchopulmonary dysplasia, *IVH* intraventricular hemorrhage, *PVL* periventricular leukomalacia, *ROP* retinopathy of prematurity.

Groups with statistically significant differences based on the Bonferroni test are indicated by superscript symbols (^*^p < 0.005 when compared with Sepsis−/NEC−, ^§^*p* < 0.005 when compared with Sepsis+/NEC−, ^¶^*p* < 0.005 when compared with Sepsis−/NEC+, ^†^*p* < 0.005 when compared with Sepsis++/NEC−, ^‡^*p* < 0.005 when compared with Sepsis+/NEC+).

Among the study population, 7139 (55.6%) infants had available data on survival status or neurodevelopmental outcomes at 18–24 months of CA. Infants without both sepsis and NEC had significantly lower frequencies of motor delay, mental delay, any NDI, and any NDI or death compared to the other groups (Table 2). In this unadjusted frequency comparison, the sepsis+/NEC− group had a higher rate of any NDI or the composite outcome of any NDI or death compared to the sepsis−/NEC− group.

**Table 2:** Neurodevelopmental outcomes of the study groups.

	Sepsis−/NEC− (*n* = 9507)	Sepsis+/NEC− (*n* = 1830)	Sepsis−/NEC+ (*n* = 536)	Sepsis++/NEC− (*n* = 533)	Sepsis+/NEC+ (*n* = 433)	*p*-value
Blindness	17/5243 (0.3%)^§^	5/947 (0.5%)^*‡^	1/229 (0.4%)^‡^	2/279 (0.7%)	4/158 (2.5%)^*§^	0.001
Hearing loss	69/4612 (1.5%)^†‡^	21/777 (2.7%)^‡^	4/209 (1.9%)^‡^	15/223 (6.7%)^*^	16/134 (11.9%)^*§¶^	<0.001
Cerebral palsy	273/5281 (5.2%)^¶†‡^	62/975 (6.4%)^†‡^	24/227 (10.6%)^*^	35/281 (12.5%)^*§^	28/160 (17.5%)^*§^	<0.001
Motor delay	648/5333 (12.2%)^§¶†‡^	155/990 (15.7%)^*¶†‡^	57/230 (24.8%)^*§^	71/290 (24.5%)^*§‡^	62/163 (38.0%)^*§†^	<0.001
Mental delay	617/3988 (15.5%)^§¶†‡^	160/726 (22.0%)^*‡^	51/183 (27.9%)^*^	59/211 (28.0%)^*^	53/129 (41.1%)^*§^	<0.001
Any NDI	1013/4083 (24.8%)^§¶†‡^	234/752 (31.1%)^*†‡^	78/189 (41.3%)^*^	102/220 (46.4%)^*§^	77/136 (56.6%)^*§^	<0.001
Any NDI or death	2030/5100 (39.8%)^§¶†‡^	545/1063 (51.3%)^*¶†‡^	253/364 (69.5%)^*§‡^	201/319 (63.0%)^*§‡^	236/295 (80.0%)^*§¶†^	<0.001

Values are expressed as *N* (%) or means ± standard deviations.

Groups with statistically significant differences based on the Bonferroni test are indicated by superscript symbols (^*^p < 0.005 when compared with Sepsis−/NEC−, ^§^*p* < 0.005 when compared with Sepsis+/NEC−, ^¶^*p* < 0.005 when compared with Sepsis−/NEC+, ^†^*p* < 0.005 when compared with Sepsis++/NEC−, ^‡^*p* < 0.005 when compared with Sepsis+/NEC+).

*Sepsis−/NEC−* No episode of sepsis or necrotizing enterocolitis (NEC), *Sepsis+/NEC−* single episode of sepsis without NEC, *Sepsis−/NEC+* NEC without sepsis, *Sepsis++/NEC−* multiple episodes of sepsis without NEC, *Sepsis+/NEC+* both sepsis and NEC, *NDI* neurodevelopmental impairment.

**Fig. 2:**
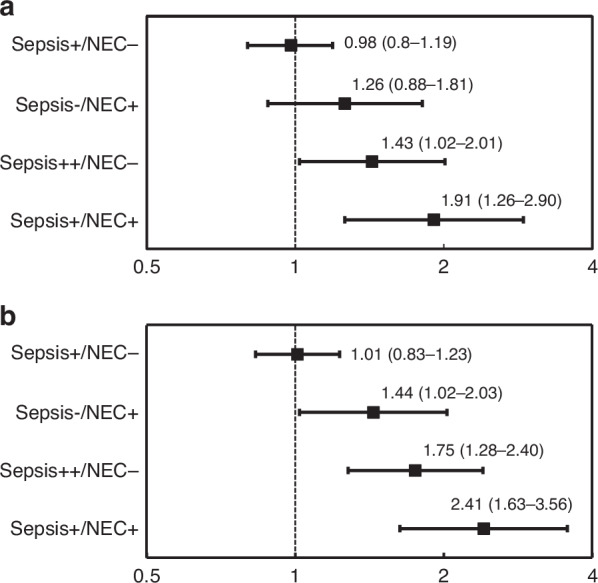
Adjusted odds ratios for neurodevelopmental impairment and the composite outcome of neurodevelopmental impairment or death. **a** Displays adjusted odds ratios (95% CI) for neurodevelopmental impairment (NDI); **b** Displays adjusted odds ratios (95% CI) for the composite outcome of NDI or death. Sepsis+/NEC− indicates a single episode of sepsis without necrotizing enterocolitis (NEC); Sepsis−/NEC+ indicates NEC without sepsis; Sepsis++/NEC− indicates multiple episodes of sepsis without NEC; and Sepsis+/NEC+ indicates both sepsis and NEC. Values are expressed as adjusted odds ratios with 95% confidence intervals.

Multivariate logistic regression analyses adjusted for potential confounders revealed that infants in the sepsis+/NEC− group had no association with any NDI and any NDI or death (Fig. 2). Infants in the sepsis−/NEC+ group showed no association with any NDI. However, they showed an association with any NDI or death. However, infants in the sepsis++/NEC− and sepsis+/NEC+ groups displayed a significantly increased risk for any NDI (adjusted odds ratio [aOR], 1.43; 95% confidence interval [CI], 1.02–2.01 and aOR, 1.91; 95% CI, 1.26–2.90, respectively) and any NDI or death (aOR, 1.75; 95% CI, 1.28–2.40 and aOR, 2.41; 95% CI, 1.63–3.56, respectively).

## Discussion

Using a nationwide registry, this study demonstrated that a single episode of sepsis without NEC was not associated with NDI at 18–24 months of CA in very preterm infants. In contrast, infants with multiple episodes of sepsis or with both sepsis and NEC were at an increased risk of NDI. The multiple-hit theory, which posits that repeated insults can lead to cumulative brain injury, is a key concept in understanding the pathophysiology of brain injury in preterm infants.^11^ Thus, distinguishing between single and multiple episodes of sepsis and/or NEC may be an important approach when examining the effects of systemic inflammation on neurodevelopmental outcomes in preterm infants.

Despite evidence linking systemic inflammation in preterm infants with adverse neurodevelopmental outcomes, several studies have reported no discernible association between sepsis and NDI in this population. A recent retrospective study involving preterm infants born at <30 weeks of gestation in the Netherlands showed that episodes of sepsis were not associated with NDI at 2 years.^8^ Another retrospective study involving VLBW infants in Japan reported that sepsis or NEC was not associated with death or NDI after discharge.^9^ A single-center cohort study involving extremely low-birth-weight infants in the US also reported that sepsis or NEC was not associated with impaired neurosensory development.^10^ However, in these studies, multiple or recurrent episodes of sepsis or NEC were not considered.

In contrast, several studies have further categorized the study population separately into those who experience both sepsis and NEC or those who experience sepsis but not NEC. A study by the NICHD Neonatal Research Network reported that infants who experienced sepsis and NEC showed the highest risk for NDI.^4^ Data from the Extremely Low Gestational Age Newborn Study revealed that developmental indices were lowest in infants who experienced bacteremia with medical or surgical NEC.^12^

Cerebral white matter injury plays a crucial role in NDI in preterm infants with sepsis or NEC.^6^ White matter injury is attributed to pre-myelinating oligodendrocytes, which are vulnerable to insults, such as hypoxia/ischemia and infection/inflammation during this period.^20^ One of the key hypotheses in white matter injury is the multiple-hit theory, which suggests that the risk of white matter injury escalates with cumulative exposure to multiple perinatal risk factors.^11,21,22^ In a study that analyzed 133 newborns born before 34 weeks of gestation, infants exposed to multiple episodes of culture-positive infections had a higher risk of progressive white matter injury on magnetic resonance imaging than those exposed to one or fewer infections.^23^

The most significant observation in this study was that infants who experienced only a single episode of sepsis did not show an increased risk of NDI. Although the detrimental effects on the white matter may accumulate with repeated systemic inflammation, a single incident may not provoke clinically discernable outcomes. As previous studies have revealed an increased risk of impaired neurodevelopment in patients with sepsis^4,5,12^ or reported no association between sepsis and neurodevelopment^9,10^ without considering the number of episodes of sepsis or NEC, the findings of the present study may have important implications. Recently, a single-center retrospective study that explored the association between coagulase-negative staphylococcal sepsis and neurodevelopmental outcomes, excluding infants with additional episodes of sepsis, showed no association between sepsis and white matter injury or adverse neurodevelopmental outcomes.^24^ Additionally, we analyzed the Sepsis+/NEC− group by categorizing patients based on the type of pathogen to investigate whether the degree of impact on NDI differs according to the infectious agent. Among the patients in this group, 1397 were infected with gram-positive bacteria, 338 with gram-negative bacteria, and 95 with fungi. When compared to the group with no events (Sepsis−/NEC−) using multivariate logistic regression, the odds ratios for NDI and NDI or death were not significantly different across all pathogen types (data not shown).

For patients who experienced NEC without sepsis, the results of the present study did not reveal an increased risk of NDI, a finding that contrasts with the prevailing view in existing research that NEC generally elevates the risk of NDI.^12,25,26^ First, these studies did not strictly exclude sepsis cases in patients with NEC. As one- or two-thirds of preterm infants with NEC also experience sepsis, it is necessary to distinguish the additional effects of sepsis to understand the effect of NEC on NDI.^12,13^ Martin et al. categorized infants with NEC with or without bacteremia and found no increased risk of NDI in infants with medical NEC without bacteremia.^12^ Second, this might be attributed to the group allocation of NEC, as we did not further categorize NEC as medical or surgical. Surgical NEC usually shows a higher degree of systemic inflammation than medical NEC, and the risk of impaired neurodevelopment is higher in surgical NEC.^26–28^ In the sub-cohort analysis, after excluding those who experienced sepsis, preterm infants with medical NEC showed no increased risk of NDI and NDI or death, whereas those with surgical NEC showed an increased risk of NDI and NDI or death (Supplementary Fig. 1).

This study had some limitations. First, although lumbar puncture is commonly performed in neonates with sepsis, the KNN database does not include specific information on whether it was conducted at the time of sepsis onset, limiting the interpretation of our findings. Cases of meningitis were excluded based solely on the reported data, which may not fully account for undiagnosed cases in the absence of lumbar puncture. Second, the follow-up rate of 55.6% at 18–24 months of CA for neurodevelopmental assessment was low, and different modalities were combined to define NDI. Furthermore, data on the severity of each event were not collected from the nationwide registry. The severity of the systemic inflammatory response experienced by patients during sepsis or NEC could vary widely, and the more severe the inflammatory response, the more significant the likelihood of white matter injury could be.^8,29^ A more detailed and insightful analysis of neurodevelopmental outcomes could have been achieved if data on inflammatory markers, detailed neuroimaging findings—including the degree of white matter injury and brain volume—and the extent of exposure to hypotension or hypoxia had been available. Moreover, genetic predisposition may affect susceptibility to inflammation or the likelihood of brain injury; however, such influences could not be accounted for in this study. However, a strength of this study is the use of a nationwide registry of preterm infants, allowing for the adjustment of various confounding factors, including prenatal inflammation, such as histologic chorioamnionitis, to analyze the association between postnatal systemic inflammation and neurodevelopmental outcomes in very preterm infants.

In conclusion, this study utilized a nationwide registry and observed that a single episode of sepsis was not associated with NDI and NDI or death. However, multiple episodes of sepsis and/or NEC significantly increased the risk of NDI and NDI or death in the study population. Although postnatal systemic inflammation was associated with adverse neurodevelopmental outcomes, a single episode of sepsis may not increase the risk of NDI in preterm infants. Further studies that would consider the degree of systemic inflammation in a single episode of sepsis and neurodevelopment are required.

## Supplementary information

Supplementary figure1

Supplementary material

## Data Availability

The datasets generated during and/or analyzed during the current study are available from the corresponding author on reasonable request.
