# Supplementary figures and images for: Adverse neurodevelopment after multiple sepsis and/or necrotizing enterocolitis in preterm infants: revisiting single-episode paradigm
Source: Pediatr Res. 2025 Jun 7;99(1):333–8. doi: 10.1038/s41390-025-04102-0 (PMC12920118; doi:10.1038/s41390-025-04102-0)

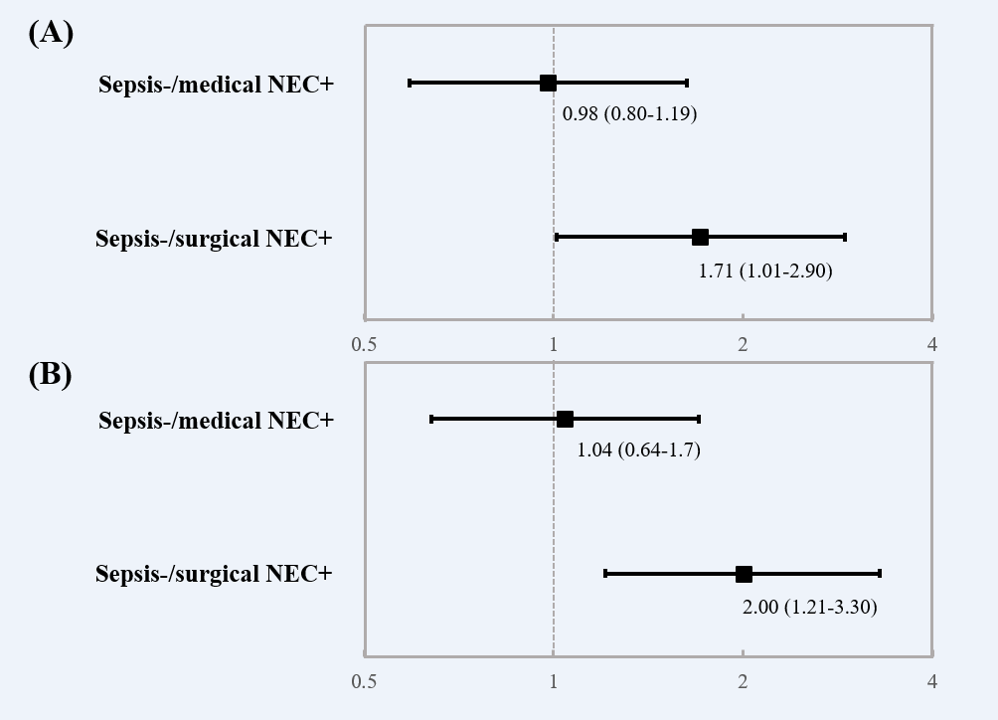

Supplement: Supplementary file 1 — Supplementary figure1 [file 41390_2025_4102_MOESM1_ESM.tif]
