# Supplementary material for: Adverse neurodevelopment after multiple sepsis and/or necrotizing enterocolitis in preterm infants: revisiting single-episode paradigm
Source: Pediatr Res. 2025 Jun 7;99(1):333–8. doi: 10.1038/s41390-025-04102-0 (PMC12920118; doi:10.1038/s41390-025-04102-0)
Supplement: Supplementary file 2 — Supplementary material [file 41390_2025_4102_MOESM2_ESM.docx]

Supplementary material

Adverse Neurodevelopment After Multiple Sepsis and/or Necrotizing Enterocolitis in Preterm Infants: Revisiting Single-Episode Paradigm

Jae Hui Ryu, M.D.^1^, Seung Han Shin, Ph.D. ^2^, Baek Sup Shin, M.D.^2^, Ee-Kyung Kim, Ph.D. ^2^, Han-Suk Kim, Ph.D. ^2^

^1^Department of Pediatrics, Ewha Womans University College of Medicine, Ewha Womans University Mokdong Hospital, Seoul, Korea

^2^Department of Pediatrics, Seoul National University College of Medicine, Seoul National University Children's Hospital, Seoul, Korea

*Corresponding Author: Seung Han Shin

Department of Pediatrics, Seoul National University Children's Hospital,

101, Daehak-ro, Jongno-gu, Seoul 03080, Korea.

Telephone number: +82-2-2072-7230

Fax number: +82-2-2072-3917

Email address: revival421@snu.ac.kr

Figure legend

Figure S1. Adjusted odds ratios of neurodevelopmental impairment (NDI) (A) and NDI or death (B) in the sub-cohort analysis or medical and surgical necrotizing enterocolitis (NEC) among infants without sepsis.

Abbreviation: Sepsis-/medical NEC= medical necrotizing NEC without sepsis; Sepsis-/surgical NEC; surgical NEC without sepsis. Values are expressed as adjusted odds ratios (95% confidence intervals).
